# The Arterial Pulse: Vascular Biology, Vascular Function Testing, and Therapies

**DOI:** 10.4061/2011/151428

**Published:** 2011-09-08

**Authors:** A. Maziar Zafari, Arshed A. Quyyumi, Julian P. J. Halcox, Sanjay Rajagopalan

**Affiliations:** ^1^Division of Cardiology, Emory University School of Medicine/Atlanta VAMC, 1639 Pierce Drive, 322 WMB, Atlanta, GA 30322, USA; ^2^Division of Cardiology, Emory University School of Medicine, 1462 Clifton Road, Suite 506, Atlanta, GA 30322, USA; ^3^Wales Heart Research Institute, Cardiff University, Heath Park Heath Park, Cardiff CF14 4XN, UK; ^4^Division of Cardiovascular Medicine, The Ohio State University Medical Center, 473 W 12th Avenue, DHLRI, Suite 200, Columbus, OH 43210, USA

In this special issue on The Arterial Pulse: Vascular Biology, Vascular Function, and Therapies, we have compiled five reviews, a clinical trial and a translational pilot study for the readership of Cardiology Research and Practice.

Techniques and approaches for modern vascular function testing have evolved over time from invasive methods restricted to smaller studies in the research laboratory to more standardized noninvasive methods, which are suitable for use in large prospective cohort studies and clinical trials. E. A. Ellins and J. P. J. Halcox describe currently available methods for the assessment of endothelial function and their potential application in cardiovascular research and clinical practice. In the second contribution E. Patvardhan and co-authors present a clinical trial investigating the relationship of the augmentation index (AIx) obtained from pressure waveforms via arterial applanation tonometry and cardiovascular risk factors and coronary artery disease (CAD). The authors conclude that AIx may be a useful measure of assessing overall risk for coronary heart disease. J. Steppan and colleagues review the effects of age-associated increase in vascular stiffness on systolic blood pressure, pulse pressure, AIx, and cardiac workload in the third paper. In this paper they describe evidence for the use of pulse wave velocity testing to measure vascular stiffness as an index of vascular health and as a predictor of adverse cardiovascular outcomes. In the fourth paper, M. Weber and colleagues present data from a pilot study about the potential diagnostic role of microRNAs (miRNAs) in blood samples of patients with angiographically documented CAD and healthy controls. The authors show that a distinct miRNA expression profile discriminates patients with CAD from healthy controls, which in turn is altered by vasoactive medications such as angiotensin-converting enzyme inhibitors and angiotensin receptor blockers. In the fifth paper, C. R. Martens and D. G. Edwards review the current literature pertaining to the potential mechanisms of peripheral vascular dysfunction in chronic kidney disease and propose possible targets for treatment. 

Vascular endothelial dysfunction is associated with a reduction in nitric oxide (NO) bioavailability, an increase in vasoconstrictors, including superoxide anions and endothelin-1 in parallel with a potential compensatory increase in other mediators of vasodilatation. This non-NO, non-prostaglandin-mediated endothelium-dependent vasodilatation has been partly attributed to endothelium-derived hyperpolarizing factors (EDHFs), the topic of the comprehensive review by M. A. Ozkor and A. A. Quyyumi who describe the role of endothelial hyperpolarization in human circulatory physiology. Finally, N. Ghasemzadeh and A. M. Zafari provide a historical review about the evolution of our knowledge of the arterial pulse from ancient times to the modern era. The authors describe the revolutionary scientific concepts and the technological innovations that provided the foundation of our current understanding of the arterial pulse as a wave that can be accurately measured with various noninvasive techniques in standardized fashion suitable for clinical patient-oriented research as well as clinical practice.

*A. Maziar Zafari* *Arshed A. Quyyumi* *Julian P. J. Halcox* *Sanjay Rajagopalan*

